# Effect of a supportive-educative nursing intervention programme on knowledge of chlorhexidine gel for umbilical cord management amongst mothers in Cross River State, Nigeria

**DOI:** 10.4102/phcfm.v13i1.2653

**Published:** 2021-04-30

**Authors:** Easter C. Osuchukwu, Chinwe F. Ezeruigbo, Paulina A. Akpan-Idiok, Ekaette F. Asuquo

**Affiliations:** 1 Department of Nursing Sciences, College of Medicine, Faculty of Allied Medical Sciences, University of Calabar, Calabar, Nigeria; 2 Department of Nursing Sciences, Faculty of Health Sciences and Technology, Ebonyi State University, Abakaliki, Nigeria

**Keywords:** supportive-educative nursing, chlorhexidine gel, umbilical cord management, Cross River

## Abstract

**Background:**

Umbilical cord infection contributes significantly to neonatal mortality rate in sub-Saharan Africa. Studies have shown low knowledge of chlorhexidine (CHX) gel for umbilical cord management amongst mothers in low-resource settings, including Nigeria.

**Objective:**

The objective of this study was to assess the effectiveness of a supportive-educative nursing intervention programme on knowledge of CHX gel amongst mothers in Cross River State, Nigeria.

**Methods:**

A quasi-experimental study design was used, and study participants comprised 168 expectant mothers, who were purposely selected and assigned to randomised control and intervention groups. The instrument for data collection was a researcher-developed structured questionnaire. The data were analysed using Statistical Package for Social Sciences version 23 for descriptive and inferential statistics at significant level was set at *p* < 0.05.

**Results:**

The result showed that at post-test the knowledge score of mothers on CHX gel improved significantly in the intervention group (*t* 77 = 24.394; *p* < 0.05). The result showed no significant difference between mothers’ demographic variables and knowledge of CHX gel.

**Conclusion:**

A supportive-educative nursing intervention programme could effectively improve knowledge of CHX gel for umbilical cord management amongst mothers. This underscores the need to improve mothers’ knowledge of CHX gel by healthcare personnel.

## Background

The global prevalence of neonatal infection remains high and accounts for one-third of neonatal deaths (1.5 million), with umbilical cord infection as the major risk factor, especially in low-income countries.^1,2^ Hospital-based studies in Nigeria revealed that umbilical cord infections account for about 10% – 19% of neonatal admissions, resulting in 30% – 49% of deaths in neonates.^3^ Nigeria therefore ranks the second highest globally, with 276 000 deaths annually resulting from umbilical cord infection.^4,5^ In Cross River State, umbilical infection is responsible for 36% of hospital admissions and 45.2% of neonatal deaths.^6^ The situation in Nigeria is also typical of other African countries like Tanzania, with neonatal sepsis accounting for 27% – 56% of neonatal mortality annually and umbilical cord infection as the major cause of infection.^7^ Umbilical cord infection causing about 30% of neonatal mortality was also reported in a related study.^8^

Although cord cleansing after delivery is viewed as mainstay of neonatal care, the substances and method of application are not consistent with the best practice guidelines.^9^ The increased neonatal mortality rate occasioned by umbilical cord infection has necessitated the recommendation of chlorhexidine (CHX) gel for cord management, which was first introduced by the World Health Organization (WHO) in Geneva on 29 September 2008 but is still poorly used in communities with high neonatal motility.^10^ This is particularly indicated for neonates during the first week of life in countries or settings with high neonatal mortality (≥ 30 neonatal deaths per 1000 live births).

Chlorhexidine gel is an antimicrobial agent that is effective against fungi, Gram-positive bacteria and Gram-negative bacteria. The bacteriostatic and bactericidal actions of CHX gel quickly destroy the cell walls of microbes and interfere with osmosis, thereby causing cell death.^7^ Specific suggestions for managing a freshly cut umbilical cord stump include hand washing, application of CHX gel and keeping the cord stump covered minimally with the baby’s usual clothing in low-resource countries.^7,11^

Achievement of compliance to the standard cord practice is far from optimal, which accounts for 40% of all deaths in children younger than 5 years annually.^5^ Various studies have identified factors that influence compliance. Evidence shows that there have been challenges adjusting to WHO guidelines on cord care in community settings as a result of poor socio-economic and sociocultural factors that are deeply rooted in complex traditional practices.^12,13^ These traditional complexities further limit women’s knowledge of standard cord care practices. Most women significantly rely on the mentorship of their grandmothers, mothers and other family members or relatives for care during pregnancy and after delivery (care of neonates).^3^

Studies suggest that home birth and application of harmful substances are common in low-income countries with elevated risk for cord infection.^13^ The level of neonatal infection is of growing concern. The high neonatal mortality rate may indicate a lack of knowledge, informing a need for focused education regarding CHX gel for cord management. Dhingra et al.^14^ reported that education of mothers on CHX at the community level showed appreciable knowledge and increased acceptance of CHX amongst mothers. Although a few studies have identified parity and lower educational status of women as major predictors of poor knowledge of CHX gel,^3,5,7^ empirical evidence on the effect of educational intervention on knowledge amongst mothers is limited in Cross River State. Thus, the purpose of this study was to determine if a supportive-educative nursing intervention programme would improve knowledge of CHX gel for cord management amongst mothers in Cross River State.

## Methods

A quasi-experimental before and after study design was adopted for the study. A multistage sampling technique was employed to recruit 168 expectant mothers in their third trimester attending prenatal care from selected primary health centres (PHCs) in Cross River State. The selection was conducted in four stages. The first stage was at the state level; Cross River State has three senatorial districts – North, Central and Southern senatorial districts. Out of these three, Southern Senatorial District was purposely selected because of its high neonatal mortality (45.2%) related to umbilical cord infection.^9^ The second stage involved the random selection (balloting with replacement) of two functional PHCs (those with delivery facilities) from each of the seven local government areas in the senatorial district, giving a total of 14 PHCs. There were a total of 19 functional primary health facilities at the time of the study. In the third stage, cluster randomisation was employed in the selection of health centres for the intervention and control groups using simple balloting (coin toss). This was done to avoid or minimise the rate of contamination and bias as done in similar studies, although the outcome is usually measured at individual level. From this exercise, seven PHCs were selected as the intervention group (PHC Ikot Offiong Ambai, PHC Ikot Edem Odo, Ediba PHC, Ikot Omin PHC, Anantigha PHC, Ekpo Abasi PHC and Atan-Onoyom PHC), whilst the other seven PHCs served as the control group (Efut Esighi PHC, Ekpri Ikang PHC, Agwagune PHC, Akpet PHC, Old Netim PHC, Awi PHC and Odukpani PHC).

Lastly, the fourth stage involved the selection of participants into the study using a purposive sampling technique with inclusion criteria and informed consent, as has been employed by some authors to conduct related studies.^6,9^ Twelve participants were selected from each of the facilities. The study was conducted at selected facilities considered for the study.

Cluster randomisation was employed in the selection of PHCs for the intervention and control groups using simple balloting (coin toss). Participants were observed before and after implementation of an intervention. The inclusion criteria included expectant mothers in the third trimester, who gave consent, were willing to deliver the baby within the study area and whose baby was born with no congenital abnormalities.

## Procedure

The study was conducted in three phases, the pretest, intervention and post-test phases. Ethical approval to conduct the study was given by the Cross River State Ethics Research Committee, Ministry of Health. Six research assistants were trained by the principal investigator on data collection techniques. Written consent was also obtained from the participants and parents of babies who were used for the study.

### First phase (prenatal)

The researcher explained the study to the participants. The participants in both the intervention and control groups had the routine prenatal health talk in the clinic and thereafter were assessed to get the baseline data (pretest) using a researcher–developed structured questionnaire, which was validated by three experts in child health. It was a three-part self-administered questionnaire requesting information on demographics and knowledge of CHX gel amongst mothers. The questionnaire was designed by the researcher and pretested amongst 20 expectant mothers who were not in the study to ascertain its reliability and validity. A computed Cronbach’s alpha coefficient of 0.79 was reported for the questionnaire.

Other research instruments included the intervention tool CHX gel, an educational plan, posters, a pamphlet and guidelines for home visits. The CHX gel educational plan was developed by the researcher with reference to WHO clinical guidelines on CHX gel. The educational package was divided into two sessions, which were mainly for the intervention, each lasting for 60 min.^5^ There was an exchange of mobile phone numbers with the participants. Each of the participants received a home visit before delivery to offer support and encouragement. The delivery dates were retrieved from the prenatal records with the help of the nurse manager.

### Second phase

This was conducted in two sessions.

#### First session

This session involved the introduction of CHX gel to the participants during the prenatal visits. Information was given on the usefulness of CHX gel in umbilical cord care. The participants were also educated on when to commence CHX gel, method of application, frequency and duration of application. Posters and samples of CHX gel were used during the sessions. There was also question and answer session.

#### Second session

This session was specifically conducted to demonstrate how to apply CHX gel shortly after severing the umbilical cord. Application of the gel was done after thorough washing of hands. Doll models were used in the demonstration. Each of the mothers took a turn to demonstrate. The duration for application of CHX gel should be 7–10 days. Pamphlets were given to the participants. The session was held at the selected health facilities considered for the study.

### Third phase

This phase took place in each participant’s house 10 days post-delivery. Each participant was visited on separate days depending on the delivery date. An assessment was conducted on their knowledge of CHX gel. Post-test data were collected using the coded questionnaire developed by the researcher for this study. The control group had no intervention, and post-test data were collected 10 days after delivery in each participant’s home based on the date of delivery. The study lasted for 12 months, May 2017 through April 2018, whilst the actual data collection lasted for 6 months.

## Method of data analysis

The study adopted both descriptive and inferential statistics in the analysis of the data generated from the study. Descriptive statistics, which included mean scores, percentages and frequency tables, were used to summarise the participants’ demographic variables as well as their knowledge of CHX gel. The study also employed Chi-square test to establish if there was a significant association between the participants’ demographic variables and their assessed knowledge of CHX gel at the pretest phase. Lastly, in order to establish a supportive-educative nursing intervention programme on knowledge of CHX gel, a paired-sample *t*-test was used to compare respondents’ assessment scores on their knowledge of CHX gel at the pretest and post-test stages for both the intervention and control groups. The significance level was set at *p* < 0.05.

### Ethical considerations

The Institution Review Board at the Cross River State Ethics Research Committee, Ministry of Health, approved the study protocol. All study participants signed a consent form prior to participation.

## Results

Table 1 shows that of the 168 respondents who participated in the study, 156 responses were documented, representing a response rate of 93%. The remaining 12 were lost during follow-up of the selected respondents. Their age ranged from < 20 to 40 years, with a mean age of 23.17 ± 7.04 years and 24.28 ± 7.79 years for the intervention and control groups, respectively. The participants were predominately of the Christian faith. Most of the participants had completed secondary education 72 (92.3%) in the intervention group and 68 (87.2%) in the control group, whilst 50 (64.1%) and 41(52.6%) participants in the intervention and control groups, respectively, lived below the nation’s minimum wage.

**TABLE 1:** Sociodemographic characteristics of the respondents.

Variable	Control group		Intervention group	
	Frequency	Percentage	Frequency	Percentage
**Age (years**)				
≤ 20	10	12.8	14	18.0
21–25	29	37.2	26	33.3
26–30	25	32.0	21	26.9
31–35	7	9.0	12	15.4
36–40	7	9.0	5	6.4
Total	78	100.0	78	100.0
**Highest educational qualification**				
BSc	13	16.7	17	21.8
HND	8	10.3	4	5.2
ND	22	28.2	26	33.3
WASC	25	32.0	25	32.0
FSLC	10	12.8	6	7.7
Total	78	100.0	78	100.0
**Number of children**				
1	32	41.0	25	32.0
2	11	14.1	23	29.5
3	15	19.2	23	29.5
4	20	25.6	7	9.0
Total	78	100.0	78	100.0
**Monthly income (rand)**				
≤ 18 000	41	52.6	50	64.1
18 001–36 000	15	19.2	9	11.5
36 001–72 000	12	15.4	8	10.3
72 001–150 000	10	12.8	11	14.1
Total	78	100.0	78	100.0

BSc, bachelor of science; HND, Higher National Diploma; ND, National Diploma; WASC, West African School Certificate; FSLC, First School Leaving Certificate.

### Knowledge of chlorhexidine amongst mothers in the control group

Table 2 presents the knowledge of CHX gel amongst the participants in the control group at pre- and post-test. At pretest 24 (30.8%) of the 78 respondents in the control group knew CHX as an antiseptic for cord care, whilst at post-test this figure increased to 30 (38.5%). The majority (27; 34.6%) did not have any idea about the antiseptic at pre- and post-test. Only 17 (21.8%) participants at pretest correctly responded to the question on when to commence CHX gel; this proportion slightly increased to 20 (25.6%) participants at post-test. At pretest, 14 (17.9%) participants knew the frequency of application of CHX gel as daily. At post-test, the number of respondents who correctly identified the frequency was 11 (14.1%). Fifteen (19.2%) participants at pretest knew that CHX is applied on the cord stump until it falls off; and this figure slightly increased to 17 (21.8%) participants at post-test. When the respondents were asked if they knew the method of application of CHX gel, only 14 (17.9%) said ‘yes’ on this item at pre- and post-test. The table further shows that 19 (24.4%) out of the 78 respondents knew that CHX gel may extend cord separation time with the use of other lotions at pretest, and at post-test this proportion increased to 23 (29.5%). Finally, at pre- and post-test, 36 (46.2%) and 37 (47.4%) respondents, respectively, correctly indicated that delayed detachment of the cord stump resulting from use of CHX cannot cause harm to the baby.

**TABLE 2:** Knowledge of chlorhexidine amongst mothers in the control group.†

Items	Pretest		Post-test	
	Frequency	Percentage	Frequency	Percentage
**What do you understand as CHX gel?**				
Cleansing solution	17	21.8	13	16.7
Antiseptic for cord care	24	30.8	30	38.5
Soap	19	24.3	21	26.9
Body lotion	18	23.1	14	17.9
Total	78	100.0	78	100.0
**Source of information**				
Internet	20	25.6	20	25.6
Health worker	14	18.0	14	18.0
Family member	16	20.5	16	20.5
Media	1	1.3	1	1.3
No idea	27	34.6	27	34.6
Total	78	100.0	78	100.0
**When to commence CHX gel**				
One week after delivery	33	42.3	31	39.7
Two days after delivery	28	35.9	27	34.6
Immediately after delivery	17	21.8	20	25.6
Total	78	100.0	78	100.0
**How often should CHX be applied to cord stump in a day?**				
Once a day	14	17.9	11	14.1
Twice a day	35	44.1	36	46.1
Thrice a day	23	29.5	24	30.8
No idea	6	7.7	7	9.0
Total	78	100.0	78	100.0
**What is the duration of CHX application (days)?**				
7–10	15	19.2	17	21.8
2	38	48.7	37	47.4
3	25	32.1	24	30.8
Total	78	100.0	78	100.0
**Do you know method of application of CHX gel?**				
Yes	14	17.9	14	17.9
No	64	82.1	64	82.1
Total	78	100.0	78	100.0
**Does CHX increase cord separating time compared to other cleansing agents?**				
Yes	19	24.4	23	29.5
No	33	42.3	31	39.7
No idea	26	33.3	24	30.8
Total	78	100.0	78	100.0
**Could delay in cord separating time cause harm to newborn?**				
Yes	16	20.5	16	20.5
No	36	46.2	37	47.4
No idea	26	33.3	25	32.1
Total	78	100.0	78	100.0

† , *n* = 78.

A summary of the control group respondents’ knowledge is presented in Table 3. At pretest, the majority (57; 73.1%) of the 78 participants in the control group had poor knowledge of CHX gel, with a mean knowledge score of 26.1190 ± 11.8519. Similarly, the majority (51; 65.4%) had poor knowledge at post-test, with a mean knowledge score of 34.476 ± 11.8387.

**TABLE 3:** Summary of knowledge of chlorhexidine gel amongst respondents in the control group.†

Assessment	Grade	Pre-test‡		Post-test§	
		Frequency	Percentage	Frequency	Percentage
≥ 77.6	Very good	7	9.0	9	11.5
57.6–77.5	Good	8	10.3	13	16.7
37.6–57.5	Fair	3	3.8	2	2.6
26.6–37.5	Poor	57	73.1	51	65.4
≤ 25.6	Very poor	3	3.8	3	3.8
Total	78	100.0	78	100.0

*Source*: Researcher’s field work, 2019

† , *n* = 78.

‡ , *u* = 26.1190; SD = 11.8519.

§ , *u* = 34.476; SD = 11.8387.

### Knowledge of chlorhexidine gel amongst mothers in the intervention group

Table 4 presents the knowledge of CHX amongst participants in the intervention group before (pretest) and after (post-test) the educational intervention. It reveals that prior to the intervention, only 13 (16.7%) participants in this group knew that CHX gel is an antiseptic for cord care, and after the educational intervention, nearly all 69 (88.4%) participants were able to identify CHX as an antiseptic for neonatal cord care. Before intervention, a greater proportion (37; 47.4%) of the 78 participants had no idea about CHX; however, after intervention the major source of information was the healthcare worker, as indicated by all 78 participants (Table 4). Only eight (10.3%) participants knew to commence usage of CHX gel immediately after delivery; this number increased to 58 (74.4%) participants after the educational intervention. On the frequency of usage of the product, only five (6.4%) respondents prior to the intervention indicated correctly that the product is applied once every day; and after the educational intervention the proportion of respondents with adequate knowledge regarding the usage frequency of the product increased to 64 (82.0%) respondents. Fifteen (19.2%) and 72 (92.3%) participants in the intervention group correctly indicated at pre- and post-test, respectively, that the product is used until the cord stump falls off. Before the intervention, only nine (11.5%) respondents knew that CHX takes days longer than other lotions to remove the cord; and after the intervention, this proportion increased to more than half (50; 64.1%) of the 78 participants. Lastly, 25 (46.2%) participants at pretest gave the correct answer by indicating that the delay in cord detachment by CHX does not cause any harm to the baby. This proportion increased to 43 (55.1%) participants after the intervention.

**TABLE 4:** Knowledge of chlorhexidine gel amongst mothers in the intervention group.†

Items	Pretest		Post-test	
	Frequency	Percentage	Frequency	Percentage
**What is CHX gel?**				
Cleansing solution	19	24.3	2	2.6
Antiseptic for cord care	13	16.7	69	88.4
Soap	22	28.2	0	0.0
Body lotion	24	30.8	7	9.0
Total	78	100.0	78	100.0
**Source of information**				
Internet	8	10.2	0	0.0
Health worker	19	24.4	78	100.0
Family member	12	15.4	0	0.0
Media	2	2.6	0	0.0
No idea	37	47.4	0	0.0
Total	78	100.0	78	100.0
**When to commence CHX gel?**				
One week after delivery	39	50.0	11	14.1
Two days after delivery	31	39.7	9	11.5
Immediately after delivery	8	10.3	58	74.4
Total	78	100.0	78	100.0
**How often should CHX be applied to cord stump in a day**				
Once	5	6.4	64	82.0
Twice	12	15.4	7	9.0
Thrice	18	23.1	7	9.0
No idea	43	55.1	0	0.0
Total	78	100.0	78	100.0
**What is the duration of CHX application (days)?**				
7–10	15	19.2	72	92.3
2	41	52.6	1	1.3
3	22	28.2	5	6.4
Total	78	100.0	78	100.0
**Do you know method of application of CHX gel?**				
Yes	10	12.8	55	70.5
No	68	87.2	23	29.5
Total	78	100.0	78	100.0
**Does CHX increase cord separating time compared to other cleansing agents?**				
Yes	9	11.5	50	64.1
No	42	53.9	13	16.7
No idea	27	34.6	15	19.2
Total	78	100.0	78	100.0
**Could cord delay in separating time cause harm to newborn?**				
Yes	36	20.5	10	12.8
No	25	46.2	43	55.1
No idea	17	33.3	25	32.1
Total	78	100.0	78	100.0

CHX, chlorhexidine.

† , *n* = 78.

A summary of respondents’ knowledge in the intervention group is presented in Table 5. According to the table, at pretest, a majority (49; 62.8%) of participants in the intervention group had very poor knowledge, with a mean knowledge score of 22.1071 ± 10.037, whilst at post-test, most (42; 53.8%) of these participants had good knowledge of CHX gel, with a mean knowledge score of 76.7738 ± 9.7131 after the educational intervention.

**TABLE 5:** Summary of knowledge of chlorhexidine gel amongst respondents in the intervention group.†

Assessment (%)	Grade	Pretest‡		Post-test§	
		Frequency	Percentage	Frequency	Percentage
≥ 77.6	Very good	5	6.4	25	32.1
57.6–77.5	Good	2	2.6	42	53.8
37.6–57.5	Fair	5	6.4	9	11.5
26.6–37.5	Poor	17	21.8	2	2.6
≤ 25.6	Very poor	49	62.8	0	0.0
Total	78	100.0	78	100.0

† , *n* = 78.

‡ , *u* = 22.1071; SD = 10.037.

§ , *u* = 76.7738; SD = 9.71318.

Table 6 indicates that the mean post-test score was higher than the mean pretest score. This observed difference was significant at *p* < 0.05. This implies that the supportive-educative nursing intervention programme was effective to upgrade the knowledge of mothers.

**TABLE 6:** Comparing the mean knowledge of chorhexidine gel among respondents in the intervention group.†

Study phase	Knowledge score for:		Statistical comparison between study groups		
	Intervention group‡	Control group‡	*t*	df	*p*
Before intervention	22.1071 ± 10.0373	26.1190 ± 11.8519	−2.281	76	< 0.05
After intervention	76.7738 ± 9.7132	34.4762 ± 11.8387	24.394	76	< 0.05

† , *N* = 78 pairs.

‡ , *n* = 78.

Table 7 indicates that none of the mothers’ sociodemographic characteristics, including monthly income, highest educational qualification, age or number of children, were significantly associated with knowledge of CHX gel.

**TABLE 7:** Chi-square test of association between mothers’ demographic characteristics and knowledge of chlorhexidine gel after intervention.†

Demographic variable	Level of knowledge					Row total	χ^2^	*p*	Remark
	Very poor	Poor	Fair	Good	Very good				
**Monthly income**									
≤ 18 000	0	1	4	19	26	50	7.20	> 0.05	Not significant
18 001–36 000	0	0	2	2	5	9			
36 001–72 000	0	1	1	2	4	8			
72 001–150 000	0	0	2	2	7	11			
Total	0	2	9	25	42	78			
**Highest educational qualification**									
BSc	0	1	2	5	9	17	12.90	> 0.05	Not significant
HND	0	0	1	0	3	4			
ND	0	0	2	9	15	26			
WASC	0	0	2	11	12	25			
FSLC	0	2	2	0	3	6			
Total	0	2	9	25	42	78			
**Age (years)**									
≤ 20	0	1	1	4	8	14	14.14	> 0.05	Not Significant
21–25	0	0	3	6	7	26			
26–30	0	1	0	9	11	21			
31–35	0	0	3	5	4	12			
36–40	0	0	2	1	2	5			
Total	0	2	9	25	42	78			
**Number of children**									
1	0	1	2	7	15	25	3.40	> 0.05	Not significant
2	0	1	4	8	10	23			
3	0	0	2	8	13	23			
4	0	0	1	2	4	7			
Total	0	2	9	25	42	78			

BSc, bachelor of science; HND, Higher National Diploma; ND, National Diploma; WASC, West African School Certificate; FSLC, First School Leaving Certificate.

† , *n* = 78.

## Discussion

The quality of care that newborns receive in the minutes after birth has a lot of influence on its survival and development. Chlorhexidine gel has been recommended by the WHO for cord management in low- and middle-income countries. However, it has been observed that there is substantial lack of empirical evidence concerning knowledge about CHX gel amongst expectant mothers in Cross River State, Nigeria. To address this gap, the researchers developed and evaluated a supportive-educative nursing intervention programme on knowledge of CHX gel amongst mothers. The effect of the supportive-educative nursing intervention programme was evaluated by comparing the mean knowledge scores between the intervention and control groups before and after the intervention. The findings, as observed in Table 3, show that there was no significant improvement in the level of knowledge about CHX gel for the control group amongst expectant mothers, but a significant improvement was reported for the intervention group (Table 5). This finding is congruent with other studies, where poor knowledge of CHX gel amongst women in non-intervention studies was reported.^15,16^ This principally implies that access to correct and factual information on CHX gel could significantly influence standard and safe cord care practices. This is evident in a study conducted in Ibadan, Nigeria, where women who had access to umbilical cord care (UCC) information exhibited good practices compared with their counterparts with limit access to information on UCC.^15^

The study further showed that there was a significant difference in knowledge scores at post-test between the intervention and control groups. This was in accordance with a study that reported a very good level of knowledge and a high mean post-test knowledge score among postnatal women after an intervention.^17^ It was observed that there was a significant difference in the mean knowledge scores between the intervention and control groups. The supportive-educative nursing intervention programme was effective at upgrading the knowledge of mothers. This indicates that giving sufficient information regarding CHX gel usage for cord management to mothers will improve their knowledge and overall neonatal care.

The study also showed in Table 7 that the sociodemographic characteristics of the participants had no significant association with post-test knowledge score in either the intervention or control groups, as reported in a similar study.^18^ This was expected, given that this was a community-based study where their behaviour was principally influenced by cultural practices and norms. Moreover, the demographic variables of the participants between the intervention and control groups were within the same range.

## Conclusion

This study showed that a well-tailored supportive - educative nursing intervention programme is significantly effective in improving expectant mothers’ knowledge of CHX gel for umbilical cord management. This can be internalised during prenatal visits. Further research should be conducted to identify possible predictors of cord care practices amongst expectant mothers.
